# Higher Socioeconomic Status Predicts Less Risk of Depression in Adolescence: Serial Mediating Roles of Social Support and Optimism

**DOI:** 10.3389/fpsyg.2020.01955

**Published:** 2020-08-06

**Authors:** Rong Zou, Xia Xu, Xiaobin Hong, Jiajin Yuan

**Affiliations:** ^1^Hubei Key Laboratory of Sport Training and Monitoring, Department of Psychology, College of Health Science, Wuhan Sports University, Wuhan, China; ^2^Institute of Brain and Psychological Science, Sichuan Normal University, Chengdu, China

**Keywords:** socioeconomic status, depression, serial-mediation model, social support, optimism, reserve capacity model

## Abstract

Family socioeconomic status (SES) is known to have a powerful influence on adolescent depression. However, the mechanisms underlying this association are unclear. Here, we explore this issue by testing the potential mediating roles of social support (interpersonal resource) and optimism (intrapersonal resource), based on the predictions of the reserve capacity model (RCM). Participants were 652 adolescents [age range: 11–20 years old, *M*_age_ = 14.55 years, *SD* = 1.82; 338 boys (51.80%)] from two junior and two senior high schools in Wuhan, China. They completed questionnaires measuring family SES, perceived social support, optimism, and depression. Results showed, as predicted, (1) SES negatively predicted adolescent depression; (2) social support and optimism serially mediated the relations between SES and depression, consistent with the predictions by the RCM. Specifically, higher SES predicted greater social support and increased optimism, which in turn contributed to reduced depression. The implications of these data to the prevention and interventions of adolescent depression were discussed.

## Introduction

As a prevalent affective disorder, depression has become the leading cause of psychophysical diseases, disability, and suicide worldwide. There are more than 264 million people of all ages suffering from depression globally (GBD Disease Injury Incidence Prevalence Collaborators, 2018). Depression often begins in adolescence since rapid biological and psychological changes during this period increase the risk of onset in depression (Hankin, 2006; Malhi and Mann, 2018). Given the typically early age-of-onset, adolescent depression has been associated both concurrently and prospectively with poor physical health and adverse psychosocial functioning (Aalto-Setälä et al., 2002; Thapar et al., 2012).

Given the high prevalence and substantial burden of depression, considerable research has focused on the factors underlying depression. As one of the fundamental environmental factors affecting many aspects of individuals’ development (Bradley and Corwyn, 2002), behavioral evidence suggests that family socioeconomic status (SES) has a powerful influence on physical and mental health, such as depression (Piko et al., 2013; Zhou et al., 2018). Neurobiological studies also show that childhood family SES predicts differences in hippocampus and amygdala volumes (Noble et al., 2012). Smaller hippocampus and amygdala volumes are associated with depression (Mervaala et al., 2000; Rosso et al., 2005). Gallo and Matthews (2003) has developed the reserve capacity model (RCM) as a theoretical framework for understanding how reserved resources, including interpersonal and intrapersonal resources, contribute to socioeconomic effects on emotional distress and physical health. However, few studies to date have explored the underlying mechanisms of the association between SES and depression both from the aspects of interpersonal and intrapersonal resources in adolescents. Exploring this issue is essential for the development of effective interventions to reduce the risk of depression in adolescents, especially those from low-SES families (Wahlbeck, 2015). Therefore, this study aimed to investigate the association between SES and adolescent depression, and more importantly, the potential underlying mechanisms based on the RCM.

### The Reserve Capacity Model

The RCM is a theoretical framework for understanding how reserve capacity mainly including interpersonal and intrapersonal resources contributes to SES-related health disparities (Gallo and Matthews, 2003). According to RCM, interpersonal resources refer to a generic protective influence associated with social functioning, emphasizing external psychosocial resources, including contact with others, network size, reciprocity in relationships, work support, and generalized social support perception (Gallo and Matthews, 2003). Meanwhile, intrapersonal resources refer to resilient intrapersonal characteristics, emphasizing internal psychosocial resources, such as self-efficacy, self-esteem, mastery, a sense of control, and optimism (Gallo and Matthews, 2003).

Studies have tested the mediating roles of reserve capacity in the associations between SES and emotional distress and physical health in adults (Gallo et al., 2005; Matthews et al., 2010). Individuals with low-SES are exposed to more daily hassles, major stressors and higher levels of chronic negative life events compared to their counterparts. These adversities may lead them not only to quick depletion of their reserves due to frequent needs to use, but also to limited opportunities to replenish and develop their resources (Gallo et al., 2005). Scarcity of resources may contribute directly to emotional distress and subsequently to health (Gallo and Matthews, 2003; Gallo et al., 2005).

However, the mediating roles of reserve capacity in the associations between SES and emotional distress and physical health were tested mainly in adults, rarely in adolescents (Gallo et al., 2005; Matthews et al., 2010). Moreover, reserve capacity has always been conceptualized as an aggregate “bank” of interpersonal and intrapersonal resources in previous studies (Gallo et al., 2005; Matthews et al., 2008). But it may be necessary and helpful to clarify the precise roles of the specific psychosocial assets in considering possible avenues for targeted intervention (Gallo, 2009). As social support represents the important part of interpersonal resources, and optimism that develops during late childhood and adolescence and thus has the potential to be intervened is closely related to adolescents’ positive adaptation (Zou et al., 2016) we chose social support and optimism to represent interpersonal and intrapersonal resources, respectively in this study, and aimed to investigate the association between SES and depression, and further to explore the precise roles of social support (interpersonal resource) and optimism (intrapersonal resource) in the SES-depression linkage among adolescents based on the RCM.

### SES, Social Support, Optimism, and Depression

#### SES and Adolescent Depression

As Bradley and Corwyn (2002) noted, as an environmental factor, SES could influence individuals’ development widely, with effects beginning prior to birth and continuing into adulthood. Regarding depression, neurobiological studies (Mervaala et al., 2000; Rosso et al., 2005; Noble et al., 2012) and most of prior behavioral studies (Piko et al., 2013; Zhou et al., 2018) have offered substantial evidence for the negative association between SES and adolescent depression, although a few studies have shown no significant association between SES and depression (Miech et al., 1999; Twenge and Nolen-Hoeksema, 2002). According to the RCM, low-SES environment is associated with greater exposure to stresses and uncertainties, which in turn, contributes to more negative emotional experiences and even emotional distress (Gallo et al., 2005). High SES may imply decreased levels of stress that contribute to the occurrence of depression (Zimmerman and Katon, 2005). Therefore, both previous studies and the RCM suggest that there would be socioeconomic disparities in adolescent depression.

#### The Mediating Role of Social Support

Family Investment Model posits that high-SES families have more resources and thus will invest more in the development of their children than low-SES families (Conger and Donnellan, 2007). While adolescents from high-SES families are afforded an array of services, goods, parental actions, and social connections, adolescents from low-SES families lack access to those same resources and experiences. With living in a low-SES environment connected to stresses, uncertainties and low-social standing, adolescents with low-SES are more likely to have poor relationships with family members, friends, and teachers (Laosa, 1977; Gallo and Matthews, 1999) which in turn make them prone to perceive less social support from family members, friends and teachers. Previous studies have provided evidence for socioeconomic disparities in social support (Schafer and Vargas, 2016).

As the important part of interpersonal psychosocial resources and a critical protective factor for adolescent development, social support has shown to be associated with many positive outcomes, such as more optimism, higher self-esteem, and higher well-being in adolescence (Chu et al., 2010; Piko et al., 2013; Olsson et al., 2016). Regarding adolescent depression, research has reached a general consensus on the benefits of social support (Rueger et al., 2016). As Matthews et al. (2010) noted, psychosocial resources could play a critical mediating role in the association between SES and health. Several studies have provided evidence for the mediating role of social support in the association between SES and physical health (Matthews et al., 2008; Richter et al., 2012). Inspired by these findings, we hypothesized that social support may mediate the SES-depression linkage.

#### The Mediating Role of Optimism

Researchers have suggested that optimism is associated both with family SES (Boehm et al., 2015; Zou et al., 2018) and with depression (Hamilton et al., 2015). Individuals from low-SES families are exposed to more stressful, threatening and demanding environments. Coping with these strains reduces these individuals’ opportunities to develop positive expectations about the future (Boehm et al., 2015; Zou et al., 2018), which in turn may have effects on their emotional states. Studies have revealed that positive expectation about the future makes optimists elicit more positive and less negative emotions and take more adaptive strategies to solve problems, which let optimists have less possibilities to experience depressive emotions (Hamilton et al., 2015). Moreover, as one component of internal intrapersonal resources and assets (Cannella et al., 2007); Zou et al. (2018) have found that optimism mediates the relation between family SES and life satisfaction in Chinese adolescents. Therefore, we hypothesized that optimism may serve as a mediator in the SES-depression linkage.

#### The Serial Mediating Roles of Social Support and Optimism

Social support is also important to many developmental processes, such as the development of optimism (Piko et al., 2013; Rueger et al., 2016). The RCM proposes that individuals with more social support have more resources to confront stress, which helps them develop a sense of control and positive expectations about the future, that is, optimism (Gallo and Matthews, 2003). This account was later confirmed by empirical studies (Segerstrom, 2007; Rueger et al., 2016). Also, positive expectations for future in turn, interfere with the development of hopelessness and depressive symptoms in adolescents (Hamilton et al., 2015). Moreover, optimism mediates the relation between social support and well-being (Karademas, 2006; Piko et al., 2013). Some research also confirms that social support serves as a mediator in the relationship of SES to personality, such as optimism (Piko et al., 2013). These evidences suggest that social support and optimism may play a serial-mediation role in the SES-depression linkage.

However, one may propose another possibility with optimism as an antecedent of social support in the serial-mediation model, given the close links between social support and optimism. Although there is evidence suggesting reciprocal relations between optimism and social support (Carver and Scheier, 2014), these studies are cross-sectional and insufficient to clarify their directions. Yet one 10-year longitudinal study demonstrates a temporal relation that only social support validly predicts optimism, but not vice versa (Segerstrom, 2007). Furthermore, social support is an important kind of external interpersonal resources related to external social functioning while optimism represents the internal psychological resources. In this regard, the hypothesis of social support as an antecedent of optimism in the serial-mediation model follows the developmental process of how external factors “get under the skin” to exert effects on internal factors (Roberts et al., 2007). Hence, according to theoretical consideration and results of the longitudinal research, we hypothesized that low-SES facilitates depression through social support and then optimism, but not the opposite direction of serial mediation.

### The Current Study

**FIGURE 1:**
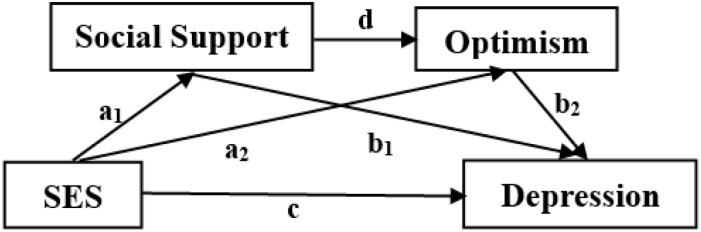
Visual representation of hypothetical model. Hypothesis 1 (H1) = total effect of socioeconomic status (SES) on depression (c); Hypothesis 2 (H2) = specific indirect effect through social support (a_1_b_1_); Hypothesis 3 (H3) = specific indirect effect through optimism (a_2_b_2_); Hypothesis 4 (H4) = serial mediating effect (a_1_db_2_).

This study aimed to test the association between SES and adolescent depression, and further to investigate the mediating effects of social support and optimism on this association based on the RCM and evidence of the relations among SES, social support, optimism, and depression. The hypotheses were (Figure 1):

H1: SES would be negatively associated with adolescent depression.

H2: Social support may play a mediating role in the SES-depression linkage.

H3: Optimism may play a mediating role in the SES-depression linkage.

H4: Social support and optimism may play a serial-mediation role in the SES-depression linkage.

## Materials and Methods

### Participants

We chose two junior and two senior high schools from a list of all high schools in Wuhan, according to the schools’ locations with different socioeconomic development (Wuhan Municipal Bureau of Statistics, 2016). Then we used the method of random cluster sampling to choose classes from each grade of each chosen school. Of the 702 possible eligible students, 652 students attending from 7th to 12th grades completed questionnaires which were checked to be valid [response rate: 92.88%; age range: 11–20 years old, *M*_age_ = 14.55 years, *SD* = 1.82; 338 boys (51.80%)]; 50 students either responded to questionnaires carelessly (answers were regular or most answers including the answers of the reverse-wording items were the same) or did not complete any of the measures because they were absent. The sex ratio approximated to that of adolescents in Wuhan (Wuhan Municipal Bureau of Statistics, 2016). No participants reported any records of using psychiatric medication. Missing data, which were less than 1% of the entire data, were estimated with expectation maximization (EM) procedure in SPSS.

### Measures

#### SES

Socioeconomic status is measured by the family affluence scale (FAS) that is well established with moderate internal consistency and good validity and widely used to measure SES among adolescents both in China and western countries (Currie et al., 2008; Liu et al., 2012). Compared to conventional indicators of family SES (e.g., educational level and occupational status of parents, family income), the FAS is much simpler and easier to answer even for young adolescents and therefore has higher completion rates (Liu et al., 2012). It includes the following four items indicating family affluence: Does your family own a car, van, or truck (0, 1, 2, or more); Do you have your own bedroom for yourself (no = 0, yes = 1); During the past 12 months, how many times did you travel away on holiday (vacation) with your family (0, 1, 2, 3, or more); How many computers does your family own (0, 1, 2, 3, or more). Scores on these four items were combined to produce a total score ranging from 0 (low affluence) to 9 (high affluence). The Cronbach’s α coefficient in this study was 0.56, which was in line with previous studies (Liu et al., 2012; Zou et al., 2016), and the McDonald’s omega was 0.64.

#### Social Support

Social support was measured by the Multidimensional Scale of Perceived Social Support (Zimet et al., 1988) that is widely used in Chinese adolescents with sufficient internal consistency and validity (Chen, 2019). It is a 12-item scale measuring perceived support from three domains: family (e.g., I get the emotional help and support I need from my family), friends (e.g., I can count on my friends when things go wrong), and significant others [e.g., There is a special person (teacher, classmate, or relative) with whom I can share joys and sorrows] on a 7-point scale, ranging from 1 = “strongly disagree” to 7 = “strongly agree,” with higher total scores implying more social support. The Cronbach’s α coefficient in this study was 0.92, and the McDonald’s omega was 0.92.

#### Optimism

Optimism was assessed by the Chinese version of the Life Orientation Test-Revised (CLOT-R; Liu and Chen, 2007). The CLOT-R has been extensively used among Chinese adolescents with good internal consistency and validity (Chen et al., 2016; Zou et al., 2018). It includes five positively worded items (e.g., When things are bad, I expect them to go better), five negatively worded items (e.g., I hardly ever expect things to go my way; reverse coded), and two filler items (e.g., It’s important for me to keep busy). Participants indicated their agreement on a 5-point scale ranging from 1 = “strongly disagree” to 5 = “strongly agree.” Total scores were built by summing up answers to the ten active items, with higher total scores representing higher optimism. The Cronbach’s α coefficient in this sample was 0.83, and the McDonald’s omega was 0.84.

#### Depression

Depression was measured by the Center for Epidemiologic Studies Depression Scale (Radloff, 1977) including 20 items. It is well validated in Chinese adolescents (Wang et al., 2013). Participants were asked how often they had been bothered by the following symptoms each item described over the last week on a 4-point scale, with higher total scores indicating more severe depressive symptoms. The Cronbach’s α coefficient in this study was 0.88, and the McDonald’s omega was 0.88.

### Procedure

Our research plan, questionnaires and the appropriate consent letters to students and their parents were submitted to the university’s Ethical Committee for Scientific Research, then examined by specialists and finally approved by the Ethical Committee. After obtaining the approval, we visited the selected school principals, handed out copies of questionnaires and consent letters, and asked for their cooperation. Then classroom teachers sent electronic consent letters to parents about the purpose of the research, the importance of the adolescents’ involvement and the voluntary nature of participation by the online Family-School Communication System and asked for parents’ support and permission. Students took home the printed consent letters that had to be signed by the students’ guardians. Classroom teachers also distributed printed consent forms to students and asked for their participation. Students with parental consent who also assented to participate were allowed to take part in the survey. A packet of self-report questionnaires was administered to students in groups of 40–50 at a time in those selected classes. Trained graduate students of psychology explained the requirements of the questionnaires using standard instructions that also stressed the anonymity of students’ identities in the survey. The average time students spent to complete the survey was approximately 15 min.

## Results

The original contributions presented in the study are publicly available. The raw data can be found here: https://doi.org/10.6084/m9.figshare.12579197.v2 (Zou et al., 2020).

### Common Method Bias Test

All data in this study were collected by the self-report method, which may lead to common method bias. So the Harman single factor test was carried out for all variables to test common method bias before data analyses (Zhou and Long, 2004). The results showed that the variance of the first factor was 27.06%, less than the critical value of 40%. Therefore, there was no serious common method bias in the data of this study.

### Preliminary Analyses

Table 1 shows univariate and bivariate statistics for all variables in this sample. Consistent with previous research (Piko et al., 2013; Zhou et al., 2018), SES was negatively correlated with depression: Adolescents with higher SES experienced less depression. Also consistent with previous studies (Boehm et al., 2015; Schafer and Vargas, 2016) SES was positively correlated with social support and optimism. Moreover, social support and optimism were both negatively correlated with depression, and positively related to each other. All correlations were in the hypothesized directions. Additionally, although prior research suggested that adolescent depression was correlated with sex and age (Hankin et al., 2015) these correlational results did not emerge in our sample. Following the principles of selecting control variables (Bernerth and Aguinis, 2016) we further tested the mediation models without including age and sex as covariates.

**TABLE 1:** Univariate and bivariate statistics for study variables.

Variables	M(SD)	1	2	3	4	5
(1) Age	14.44 (1.82)	–				
(2) Sex	0.48 (0.50)	–	–			
(3) SES	4.61 (2.05)	−0.25***	0.11**	–		
(4) SS	58.66 (13.40)	0.04	0.08	0.16***	–	
(5) OP	34.28 (7.09)	–0.06	–0.02	0.18***	0.56***	–
(6) DE	16.46 (10.64)	0.05	–0.02	−0.16***	−0.53***	−0.64***

*N* = 652; M, mean; SD, standard deviation; SS, social support; OP, optimism; DE, depression; SES, socioeconomic status. Sex was dummy coded such that boys = 0, girls = 1. **p < 0.01, ***p < 0.001.

### Mediation Analyses

To test the multiple serial-mediation hypotheses, we used the SPSS macro PROCESS (Hayes, 2013) which has been used by lots of researchers (Tan et al., 2018). The Model 6 with 95% bias-corrected confidence intervals (CIs) based on 10,000 bootstrap samples was used to examine the indirect effects of SES on depression through social support and optimism. An effect is considered to be statistically significant at *p* = 0.05 if the 95% CI does not include zero. To reduce multicollinearity and yield standardized coefficients, all variables were *z*-score transformed prior to analysis.

Results (see Table 2) showed that SES significantly predicted social support (β = 0.16, *p* < 0.001) and optimism (β = 0.09, *p* < 0.05). Social support was significantly associated with optimism (β = 0.55, *p* < 0.001). Both Social support and optimism were significantly related to depression (β = −0.24, *p* < 0.001 for social support; β = −0.49, *p* < 0.001 for optimism).

**TABLE 2:** Regression results for the serial-mediation model.

Antecedent	Consequent								
	SS			OP			DE		
	β	SE	95%CI	β	SE	95%CI	β	SE	95%CI
**SES**	0.16	0.04	**[0.08, 0.24]**	0.09	0.03	**[0.02, 0.16]**	−0.03	0.03	[−0.10, 0.03]
**SS**	–	–	–	0.55	0.04	**[0.47, 0.63]**	−0.24	0.04	**[−0.33, −0.16]**
**OP**	–	–	–	–	–	–	−0.49	0.04	**[−0.57, −0.42]**
	*R*^2^ = 0.03			*R*^2^ = 0.32			*R*^2^ = 0.45		
	*F*_(3,648)_ = 14.88***			*F*_(4,647)_ = 104.51***			*F*_(5,646)_ = 177.72***		

**N* = 652. Bolded confidence intervals do not include a zero, indicating a significant effect. SS, social support; OP, optimism; DE, depression; SES, socioeconomic status; CI, confidence interval.*

**FIGURE 2:**
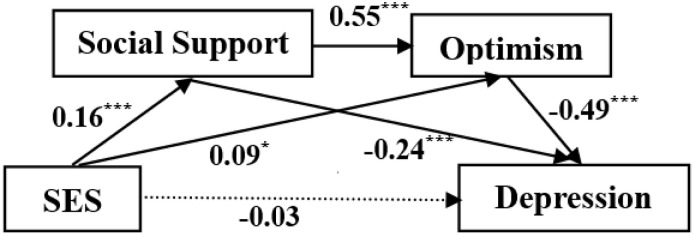
Serial-mediation model showing effects of socioeconomic status (SES), social support, and optimism on depression. Standardized coefficients were presented. The dotted line denotes insignificant direct effect from SES to depression. *p < 0.05, ***p < 0.001.

Further analysis (see Table 3) showed that total effects of SES on depression were significant (effect = −0.16, 95% CI [−0.23, −0.09]), indicating socioeconomic inequalities in depression. Meanwhile, the total indirect effects were significant (effect = 0.13, 95% CI [−0.18, −0.07]), which implied that social support and optimism mediated the SES-depression linkage. However, the direct effect of SES on depression did not emerge after controlling the impacts of social support and optimism (effect = −0.03, 95% CI [−0.10, 0.03]), which indicated that as a distal factor, the effect of SES on adolescent depression was fully mediated by proximal factors (i.e., social support and optimism; Figure 2).

**TABLE 3:** Effects of SES on depression.

	Effect Size	Boot SE	Boot CI
Total effects	−0.16	0.04	**[**−**0.23,** −**0.09]**
Direct effect	−0.03	0.03	[−0.10, 0.03]
Total indirect effects	−0.13	0.03	**[**−**0.18,** −**0.07]**
Indp1: SES→SS→DE	−0.04	0.01	**[**−**0.07,** −**0.02]**
Indp2: SES→SS→OP→DE	−0.04	0.01	**[**−**0.07,** −**0.02]**
Indp3: SES→OP→DE	−0.04	0.02	**[**−**0.08,** −**0.01]**

*Indp, Indirect path; SS, social support; OP, optimism; DE, depression; SES, socioeconomic status; CI, confidence interval. Bolded confidence intervals do not include a zero, indicating a significant effect.*

As predicted, all the three hypothetical mediating pathways were supported (see Table 3). Social support and optimism independently and jointly mediated the SES-depression linkage, effect = −0.04, 95% CI [−0.07, −0.02] for social support; effect = −0.04, 95% CI [−0.08, −0.01] for optimism; effect = −0.04, 95% CI [−0.07, −0.02] for social support and then optimism (i.e., serial multiple mediation).

## Discussion

This study tested the relationship between SES and depression and further explored the possible pathways underlying this association in terms of both interpersonal and intrapersonal resources among Chinese adolescents. Understanding these socioeconomic inequalities in adolescent depression and the underlying psychological mechanisms is essential for effective interventions to reduce the risk of adolescent depression. Our results showed a significantly negative association between SES and adolescent depression, with the association being mediated both independently and accumulatively (i.e., serial mediation) by social support and optimism. The strengths of this study can be summarized by three points. (1) Integrating previous studies that only examined the role of either social support or optimism in the relationship between SES and depression, we simultaneously took into account SES (objective environment), social support (interpersonal context), optimism (intrapersonal resource), and depression (mental health) to offer a comprehensive picture of the SES-depression linkage. The serial-mediation model we found offers new insights into our understanding about how objective environments “get under the skin” to exert effects on interpersonal contexts and then intrapersonal resources, and how intrapersonal resources “get outside the skin” to have effects on mental health (Roberts et al., 2007). (2) We clarify the precise roles of the specific psychosocial assets that have always been conceptualized as an aggregate “bank” of resources in previous studies, which is necessary and valuable in considering possible avenues for targeted intervention (Gallo, 2009). (3) Considering the close relationship between depression and physical health, these findings also provide one possible path and explanation for the robust association between SES and physical health both from perspectives of reserved resources and negative emotions.

This study revealed several meaningful findings. First, as hypothesized, SES was negatively associated with adolescent depression. It is consistent with previous studies implying that adolescents with lower SES have greater risk of depression relative to their counterparts (Piko et al., 2013; Zhou et al., 2018). Although a few studies indicated no significant association between SES and depression (Miech et al., 1999; Twenge and Nolen-Hoeksema, 2002), the majority of evidence suggests that socioeconomically disadvantaged adolescents are more likely to develop depression. Our results lend further evidence for those low-SES environments are predictive for adolescent depression.

Second, consistent with the RCM and our hypotheses, social support and optimism independently mediated the SES-depression linkage. According to the RCM, low-SES connecting to stresses and uncertainties depletes reserves, which in turn exerts effects on emotional distress or even depression (Gallo and Matthews, 2003). Our results showed that SES could predict social support and optimism, and that both social support and optimism were predictive of adolescent depression. Moreover, our data supported that SES was associated with depression indirectly via social support and optimism, respectively. These findings suggest that SES seems to operate largely through both interpersonal and intrapersonal resources to affect depression.

Finally, as hypothesized, the serial-mediation model with social support as an antecedent of optimism was found. The result that social support was an antecedent factor is consistent with previous studies indicating that the growth of social support may give people a more positive view of their future and increase optimism (Karademas, 2006; Segerstrom, 2007). Specifically, the serial-mediation findings show that SES is indirectly associated with adolescent depression through social support and then optimism. Consistent with the RCM, low-SES denotes reduced social support during development, which in turn contributes to low levels of optimism during interpretation of environmental stressors, consequently correlating with enhanced risk of depression. Additionally, while we simultaneously took into account SES (objective environment), social support (interpersonal context), optimism (intrapersonal resource), and depression (mental health) to offer a comprehensive picture of the SES-depression linkage, the serial-mediation model offers new insights into our understanding about how objective environments “get under the skin” to exert effects on interpersonal contexts and then intrapersonal resources, and how psychological resources “get outside the skin” to exert effects on mental health (Roberts et al., 2007).

Just from statistical point of view, another serial-mediation model with optimism as an antecedent of social support can be supported (see Supplementary Figure S1). However, combining the theoretical consideration about the logical relationship between external and internal factors (Roberts et al., 2007) with results of the longitudinal research that only social support validly predicts optimism, but not vice versa (Segerstrom, 2007) it is more reasonable to follow the hypothesized serial-mediation model where low-SES facilitates depression through social support and then optimism. Nevertheless, as Fried (2020) proposed, the fact that the current data was not able to distinguish these two models statistically also reminds us that we need stronger theories that provide more precise predictions.

This study helps us better understand the significant role of SES in adolescent depression and the underlying mediating mechanisms of this association. It also provides evidence for the RCM. Moreover, while adverse effects of low-SES on adolescents’ mental health have proven an intractable problem worldwide thus far, our study provides important practical implications to this issue. Despite the best efforts of policy makers to provide low-SES families with material resources and skills necessary to ameliorate adverse social and environmental conditions, these assistances are inadequate at preventing a significant translation of socioeconomic inequalities into health inequalities (Zimmerman and Katon, 2005). As stress may also mediate the effects of SES on depression (Patel et al., 2018) considering the important roles of social support and optimism in the SES-depression linkage, intervention programs should focus on promoting adolescents’ social support and the development of their optimism, together with efforts to reduce hassles and source of chronic stress for adolescents, may help make a difference in improving the mental health of adolescents confronting disadvantageous circumstances in early life.

### Limitations

First, using a cross-sectional design in this exploratory study prohibited conclusion on temporal relations between these variables. Although the current study is based on the RCM and many previous studies, future research is needed using a longitudinal approach to further validate the direction from social support to optimism, and draw a better picture of how SES correlates adolescent depression over time. The second limitation is that the data are simply based on adolescents’ self-reports and the findings are prone to mono-method bias. Multiple-source measurements should be used in further studies. Finally, we must acknowledge that as stress may account for much of the difference in developmental outcomes between low-SES and high-SES individuals (Bradley and Corwyn, 2002; Gallo and Matthews, 2003) it would have been more logical and rigorous to also include in the model a measure of stress that we ignored in this study. Further studies should incorporate stress into the model to specify multiple mediating mechanisms linking SES and adolescents’ depression.

### Conclusion

The current study observed that SES negatively predicted adolescent depression by the serial mediation of social support (interpersonal resource) and optimism (intrapersonal resource), consistent with the predictions by the RCM. Specifically, higher SES predicted greater social support and increased optimism, which in turn contributed to reduced depression. These data contribute to our understanding of how family SES operates through psychosocial processes and consequently contribute to the incidence of adolescent depression. Along with other research in the area, these findings provide empirical evidence for the development of adolescent depression, and also suggest possible avenues for prevention and intervention efforts.

## Data Availability Statement

The raw data can be found here: https://doi.org/10.6084/m9.figshare.12579197.v2 (Zou et al., 2020).

## Ethics Statement

The studies involving human participants were reviewed and approved by Ethical Committee for Scientific Research of Sichuan Normal University. Written informed consent to participate in this study was provided by the participants’ legal guardian/next of kin.

## Author Contributions

RZ: conceptualization, funding acquisition, writing – original draft, data curation, methodology, and formal analysis. XX: conceptualization, funding acquisition, writing – original draft, and review and editing. XH: conceptualization, funding acquisition, and writing – review and editing. JY: conceptualization, funding acquisition, writing – original draft, data curation, methodology, and review and editing. All authors contributed to the article and approved the submitted version.

## Conflict of Interest

The authors declare that the research was conducted in the absence of any commercial or financial relationships that could be construed as a potential conflict of interest.
