# Supplementary material for: Higher Socioeconomic Status Predicts Less Risk of Depression in Adolescence: Serial Mediating Roles of Social Support and Optimism
Source: Front Psychol. 2020 Aug 6;11:1955. doi: 10.3389/fpsyg.2020.01955 (PMC7425112; doi:10.3389/fpsyg.2020.01955)
Supplement: Supplementary file 1 [file Image_1.pdf]

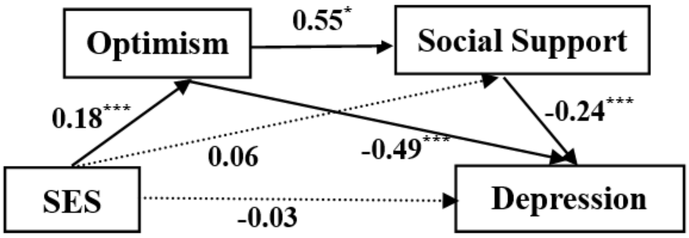

FIGURE S1 Another Serial-mediation model with optimism as an antecedent of social support. Standardized coefficients were presented. The dotted lines denote insignificant direct effect from SES to depression and insignificant indirect effect of socia support in the SES-depession linkage. $^{*}p < 0.05$, $^{***}p < 0.001$.
